# Transcriptional Profiling of Human Familial Longevity Indicates a Role for *ASF1A* and *IL7R*

**DOI:** 10.1371/journal.pone.0027759

**Published:** 2012-01-11

**Authors:** Willemijn M. Passtoors, Judith M. Boer, Jelle J. Goeman, Erik B. van den Akker, Joris Deelen, Bas J. Zwaan, Ann Scarborough, Ruud van der Breggen, Rolf H. A. M. Vossen, Jeanine J. Houwing-Duistermaat, Gert Jan B. van Ommen, Rudi G. J. Westendorp, Diana van Heemst, Anton J. M. de Craen, Andrew J. White, David A. Gunn, Marian Beekman, P. Eline Slagboom

**Affiliations:** 1 Section of Molecular Epidemiology, Leiden University Medical Center, Leiden, The Netherlands; 2 Center for Human and Clinical Genetics, Leiden University Medical Center, Leiden, The Netherlands; 3 Department of Medical Statistics, Leiden University Medical Center, Leiden, The Netherlands; 4 Information and Communication Theory Group – Bioinformatics, Faculty of Electrical Engineering, Mathematics and Computer Science, Delft University of Technology, Delft, The Netherlands; 5 Section of Evolutionary Biology, Institute for Biology, Leiden University, Leiden, The Netherlands; 6 Measurement Science, Unilever Colworth Laboratory, Sharnbrook, Bedfordshire, United Kingdom; 7 Leiden Genome Technology Center, Leiden University Medical Center, Leiden, The Netherlands; 8 Department of Gerontology and Geriatrics, Leiden University Medical Center, Leiden, The Netherlands; 9 Safety Environmental Assurance Centre, Unilever Colworth Laboratory, Sharnbrook, Bedfordshire, United Kingdom; 10 Unilever Discover, Colworth, Sharnbrook, Bedfordshire, United Kingdom; 11 Netherlands Consortium for Healthy Ageing, Leiden, The Netherlands; Universität Heidelberg, Germany

## Abstract

The Leiden Longevity Study consists of families that express extended survival across generations, decreased morbidity in middle-age, and beneficial metabolic profiles. To identify which pathways drive this complex phenotype of familial longevity and healthy aging, we performed a genome-wide gene expression study within this cohort to screen for mRNAs whose expression changes with age and associates with longevity. We first compared gene expression profiles from whole blood samples between 50 nonagenarians and 50 middle-aged controls, resulting in identification of 2,953 probes that associated with age. Next, we determined which of these probes associated with longevity by comparing the offspring of the nonagenarians (50 subjects) and the middle-aged controls. The expression of 360 probes was found to change differentially with age in members of the long-lived families. In a RT-qPCR replication experiment utilizing 312 controls, 332 offspring and 79 nonagenarians, we confirmed a nonagenarian specific expression profile for 21 genes out of 25 tested. Since only some of the offspring will have inherited the beneficial longevity profile from their long-lived parents, the contrast between offspring and controls is expected to be weak. Despite this dilution of the longevity effects, reduced expression levels of two genes, *ASF1A* and *IL7R*, involved in maintenance of chromatin structure and the immune system, associated with familial longevity already in middle-age. The size of this association increased when controls were compared to a subfraction of the offspring that had the highest probability to age healthily and become long-lived according to beneficial metabolic parameters. In conclusion, an “aging-signature” formed of 21 genes was identified, of which reduced expression of *ASF1A* and *IL7R* marked familial longevity already in middle-age. This indicates that expression changes of genes involved in metabolism, epigenetic control and immune function occur as a function of age, and some of these, like *ASF1A* and *IL7R*, represent early features of familial longevity and healthy ageing.

## Introduction

Nonagenarians and centenarians delay or escape age-related diseases [1], their first degree family members have a life-long survival advantage [2], [3] and their middle-aged offspring have a decreased prevalence of and mortality from coronary heart disease, type 2 diabetes, and cancer [4], [5]. In addition, the offspring of long-lived individuals have beneficial physiological characteristics for lipid and lipoprotein particle profiles [6], [7], glucose metabolism and insulin sensitivity [8], [9]. However, they do not differ from controls with respect to body mass index, serum IGF-1 levels, height and lifestyle factors such as physical activity levels and smoking behavior [10], [11]. Although familial longevity is a complex phenotype, identifying transcriptional targets that may contribute to the physiological benefits observed in long-lived families will increase our understanding of which pathways can influence susceptibility to and protection from age-related disease.

Previous studies have investigated whether there are gene expression changes that occur with age in brain, lymphocyte, kidney and skeletal muscle tissues [12]–[17]. The expression of some genes were found not only to change with age but also to reflect the biological function of the source organs [18], [19]. These studies have demonstrated that gene expression levels are not only markers of chronological age but also of tissue function. However, these studies cannot discriminate between genes showing expression changes in mid-life that may contribute to the aging process, from those showing expression changes later in life as a consequence of the aging process.

Here we report transcriptional profiling of whole blood samples from participants of the Leiden Longevity Study which is based in the Netherlands and comprises nonagenarian sibling pairs, their middle-aged offspring and the partners of the offspring as population controls. A comparison of gene expression profiles between the nonagenarian siblings and controls identified profiles that associate with age. The subsequent comparison of the middle-aged offspring and the controls enabled the identification of genes that mark the propensity to become long-lived in middle-age.

## Results

### Whole genome microarrays and analysis design

Gene expression profiles were generated from 150 whole blood total RNA samples collected from 50 families belonging to the Leiden Longevity Study (LLS). From each family, one nonagenarian sibling, one of their offspring and the offspring's partner (Table 1) were profiled. We identified 47,209 probes (88% of the total number of probes) which were expressed in at least 10% of the samples. Of these, 45,164 probes (containing at least 17,896 unique genes) could be mapped to a chromosomal position and were, therefore, used for further analyses.

**Table 1:** Description of the participants of the LLS in the microarray and validation population.

		Controls	Offspring	Nonagenarians
A	*Microarray experiment*			
	N	50	50	50
	Males/females (% males)	24/26 (48%)	25/25 (50%)	26/24 (52%)
	Mean age in years (range)	61.9 (43.7–78.8)	60.8 (42.8–74.8)	93.4 (89.3–102.2)
B	*RT-qPCR experiment*			
	N	312	332	79
	Males/females (% males)	143/169 (45.8%)	190/142 (57.2%)	34/45 (43.0%)
	Mean age in years (range)	61.3 (40.9–81.4)	61.3 (33.6–78.3)	94.1 (89.0–101.2)

**Figure 1:**
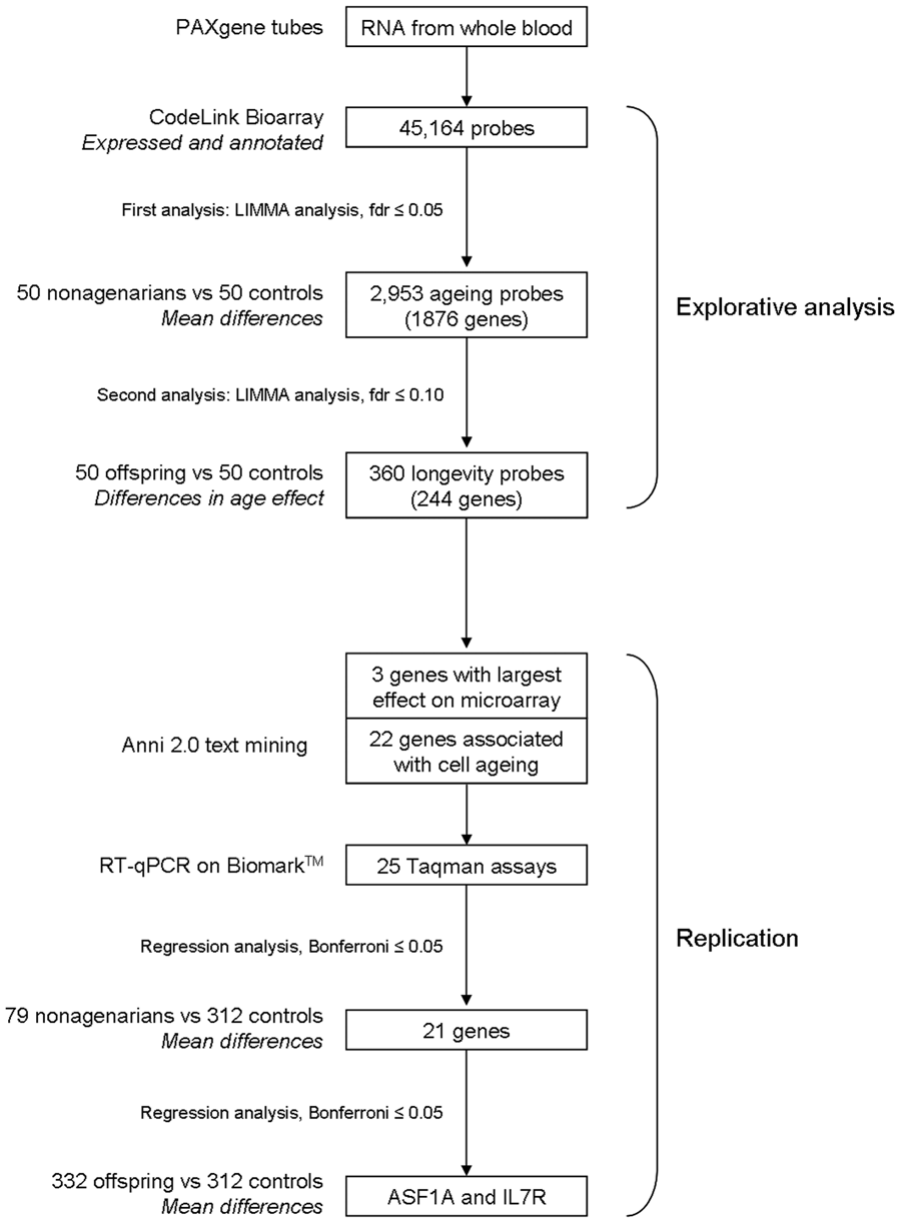
Flowchart of gene expression analyses. The order of analyses is shown for the explorative analysis (top half of the figure) and the replication analysis (bottom half of the figure). The probes/genes are depicted in the boxes and to the left thereof are the techniques or analyses used.

The explorative analysis is divided in two parts (Figure 1). The first analysis focused on the comparison between the long-lived nonagenarians and the population controls (the partners of the offspring). Using this design we aimed to find genes whose expression changed with increasing age and among these, those that were differentially expressed in long-lived families. In the second analysis we investigated which of the differentially expressed genes emerging from the first analysis were already differentially expressed between long-lived family members and controls in middle-age. Therefore we compared the offspring to the controls for mean gene expression differences and also for the interaction between the two groups with age to identify genes whose expression changed differentially with age between offspring and controls. Following the explorative analysis, we performed replication analyses in an extended group of the LLS using RT-qPCR on a selected subset of the differentially expressed genes.

### Differential gene expression associating with age

**Figure 2:**
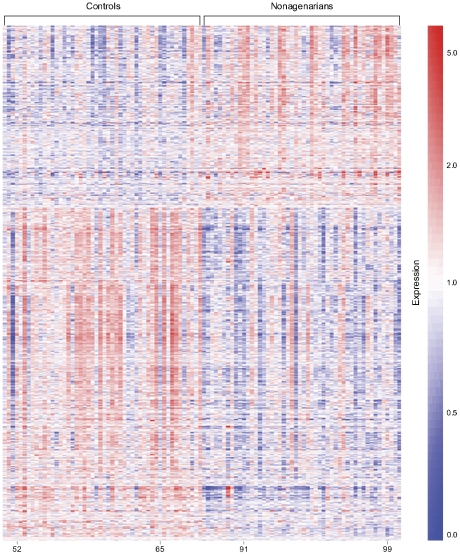
Expression profiles of 2,953 probes that differed between nonagenarians and middle-aged controls. Expression intensities of the 2,953 probes were analyzed by one-dimensional hierarchical clustering. Each probe is represented by a row; each subject by a column. Samples are organized left to right by increasing age which is indicated for a few individuals for reference. The largest cluster of probes exhibits reduced expression (transition from red to blue), and another cluster exhibits increased expression (transition from blue to red) in nonagenarians compared to controls. Mean centered expression values of probes are displayed according to the color scale in which red represents above average expression levels and blue below average expression levels. Fold changes of individual probes are given in Supplementary Table S1.

To investigate the differences in gene expression levels between the nonagenarian subjects and the middle-aged controls a linear regression model was applied to the probe data. With adjustments for gender and batch effects (Model 1, see Materials and Methods) 2,953 probes (of which 1,853 represented known genes) were found to be differentially expressed at a false discovery rate (FDR) of 0.05 (Figure 2 and Supplementary Table S1). The expression levels of 1,046 probes were increased and 1,907 decreased in the nonagenarians compared to the younger controls. The probe that associated with age with the highest significance (FDR adjusted p-value = 5×10^−10^) is located in the leucine rich repeat neuronal 3 gene (*LRRN3*, 7q31.1) and showed a 3.1-fold decreased expression in nonagenarians, which was also the largest difference in expression level between the two groups. The largest increase in expression level was 2-fold for a probe targeting the interferon, alpha-inducible protein 27 isoform (*IFI27*, 14q32.13) gene locus. This probe could also target *SYTL1*, but since the levels of a specific probe for *SYTL1* did not correlate with the *IFI27* probe (Pearson correlation = −0.14), while a unique probe for *IFI27* did correlate (Pearson correlation = 0.96), the result suggests that *IFI27* mRNA is responsible for the association. Average expression levels of the 2,953 probes show a distribution over the whole range of measured intensities (Supplementary Figure S1) and 89.5% of these probes were detected in ≥95% of the samples.

If a group of differentially expressed genes act in the same pathway or biological function this could lead to an additive or even synergistic effect on cellular function. Furthermore, the interpretation of transcriptional data in a pathway context is a more robust signal to noise measurement. The Gene Ontology (GO) consortium [20] provides structured vocabularies and classification of genes, covering several domains of molecular and cellular biology. Hence, GO terms were tested for differences between nonagenarians and controls using the Globaltest methodology [21], [22]. Globaltest determines whether the expression pattern of genes within a set as a whole is associated with an outcome, in this case being nonagenarian or not, without testing single probes. We assayed 1,808 GO gene sets, representing groups of genes closely related in their biological function or process, containing at least 10 probes per GO term, again using Model 1. The Globaltest showed that 109 GO term gene sets were significantly differentially expressed between nonagenarians and controls at a family-wise error rate (FWER) of ≤0.05 (Supplementary Table S2). FWER is used because the gene sets for GO terms are partly overlapping and therefore not independent. These 109 GO terms include 73 biological processes, 31 molecular functions and 5 cellular components. The biological processes identified under the higher level GO classifications were ‘lymphocyte activation’, ‘anatomical structure development’, ‘response to stimulus’ (including ‘immune response’), ‘regulation of gene expression’, and ‘regulation of signal transduction’. For the molecular functions list, pathways involved in protein binding were the most abundant.

### Differential gene expression in middle-age associating with longevity

The nonagenarian participants in the LLS each exhibit the longevity phenotype and their offspring, as a group, carry the potential to become long-lived as demonstrated by beneficial physiological characteristics and decreased morbidity of that generation [23]–[25]. The physiological differences between offspring and controls are small since not all individual offspring may have inherited the longevity trait. To distinguish which of the 2,953 probes that associated with age also reflect familial longevity already in middle-age, gene expression levels were compared between the 50 offspring of the nonagenarians and the 50 controls (Table 1). In a linear regression model adjusting for age, gender and batch effects (Model 2, see Materials and Methods), we observed no significant differences in the average expression levels between the offspring and controls. Next, we investigated differential expression changes as a function of age between the offspring and the controls by testing the interaction between group and age (Model 3, see Materials and Methods). Differential expression (FDR≤0.1) between offspring and controls across the age-range (43–79 years) was observed for 360 probes, representing 244 unique genes (Supplementary Table S3). Of these probes, 359 had a fold change below 1, indicating that expression levels had either a weaker increase with age or a stronger decrease with age in the offspring compared to controls. These age-related expression differences may represent early characteristics of human longevity.

The most significant differentially expressed probe (FDR adjusted p-value = 0.050, 1.6-fold decrease every ten years) corresponded to the zinc finger protein 331 gene (*ZNF331*, 19q13.33) whereas the largest decrease in expression level was for a probe targeting a mRNA at chromosome 1q43 (no known corresponding gene); this had a 2.1-fold decreased expression in offspring every ten years relative to controls. The only probe that demonstrated a significant increase in expression with age in the offspring (a 1.3-fold change every ten years) targeted an EST at 20q13.2 (no known corresponding gene, NCBI Build 36). We observed no differential expression between offspring and controls for *LRRN3* and *IFI27*, which had the most extreme changes with age in the first analysis in which nonagenarians and controls were compared.

To investigate differential expression changes with age in a set of genes acting in the same pathway or having the same function, we implemented Model 3 in GlobalAncova [26]; a method similar to the Globaltest for GO terms but additionally suited for models including interaction terms. The GO biological process that was most prominent among the longevity associated gene expression profile was the Rho protein signal transduction pathway (GO:0007266) (FWER = 0.079).

### Extensive replication study

To replicate our results we measured the expression levels of a subset of genes via RT-qPCR in 79 nonagenarians, 332 offspring and 312 controls (Table 1 and Materials and Methods). We first selected two genes on the basis of their effect size and significance in the analysis between nonagenarians and controls (i.e. *LRRN3* and *IFI27*) and the gene different between offspring and controls (i.e. *ZNF331*). We added 22 genes that were differentially expressed in the offspring compared to controls which additionally associated with the concept “cell aging” in literature using the text-mining tool Anni 2.0 [27], http://biosemantics.org/anni/. In total 25 genes (Figure 1), including the *WRN* progeria gene, the *MYC* cancer gene and the longevity *MTOR* (also known as *FRAP1*) gene, were selected for replication analyses.

As replication of the first analysis, the comparison between nonagenarians and controls, the expression of 21 out of 25 genes was in concordance with the observations in the microarray dataset and hence the RT-qPCR results confirmed the microarray findings (Table 2). *LRRN3* again showed the largest significant decreased expression in the nonagenarian siblings and *IFI27* the largest increased expression. The analysis to test for replication of the novel samples only (58 nonagenarians and 281controls) resulted in the same observations (Supplementary Table S4).

**Table 2:** RT-qPCR results.

			Nonagenarians vs. controls		Offspring vs. controls	
		N =	79	312	332	312
Gene name		Assay	FC	p	FC	p
	*Top genes*					
1	IFI27	Hs01086373_g1	1.41	**2.6×10^−4^**	NA	NA
2	LRRN3	Hs00539582_s1	0.56	**<10^−6^**	NA	NA
3	ZNF331	Hs00218578_m1	0.93	**1.0×10^−4^**	0.99	0.574
	*Cell aging associated genes*					
4	ADAMTS5	Hs00199841_m1	1.01	0.008	1.00	0.872
5	ASF1A	Hs01011627_m1	0.85	**<10^−6^**	0.88	**0.002**
6	CCR6	Hs01890706_s1	0.68	**<10^−6^**	1.06	0.265
7	CD248	Hs00535586_s1	>10	0.322	1.04	0.736
8	CDK6	Hs00608037_m1	0.95	0.053	0.95	0.015
9	ENO2	Hs00157360_m1	0.99	**<10^−6^**	0.99	0.044
10	FLT3LG	Hs00181740_m1	0.25	0.029	0.63	0.202
11	HK3	Hs01092843_g1	1.17	**<10^−6^**	1.00	0.929
12	IL7R	Hs00902338_g1	0.76	**<10^−6^**	0.89	**0.001**
13	LEF1	AI6Q1P7	0.64	**<10^−6^**	0.97	0.565
14	MLLT3	Hs00971090_m1	0.80	**<10^−6^**	0.95	0.087
15	MTOR (FRAP1)	Hs00234508_m1	0.97	**6.0×10^−6^**	0.99	0.337
16	MYC	Hs00153408_m1	0.78	**<10^−6^**	0.98	0.547
17	NOLC1	Hs01102319_g1	0.89	**1.0×10^−6^**	0.95	0.082
18	NR3C2	Hs00230906_m1	0.78	**<10^−6^**	0.95	0.059
19	RUVBL2	AI7ZZWF	0.79	**6.0×10^−5^**	1.07	0.469
20	SIDT1	Hs00214475_m1	0.70	**5.0×10^−6^**	0.91	0.301
21	SMAD3	Hs00706299_s1	0.87	**<10^−6^**	0.99	0.825
22	SMYD5	Hs00300181_m1	0.93	**<10^−6^**	0.98	0.229
23	TCF12	Hs00918972_m1	0.90	**<10^−6^**	0.97	0.127
24	TCF4	Hs00972428_g1	0.88	**1.5×10^−5^**	1.01	0.872
25	WRN	Hs02561119_s1	0.76	**<10^−6^**	1.02	0.791

FC: fold change between groups; a FC above 1 indicates an increase in expression and a FC below 1 indicates a decrease in expression compared to the controls. p: unadjusted p values. Bold indicate p values are below the significant level of 0.05 after Bonferroni correction for multiple testing (threshold p = 0.002).

To identify longevity associated genes in middle-age, we replicated the second analysis by comparing the mean expression levels of 23 genes between the offspring of the nonagenarians and the controls (Model 2, see Materials & Methods). We excluded *LRRN3* and *IFI27* from this analysis since expression of these two genes was not different between offspring and controls in the microarray dataset. Two genes showed significant differential expression after Bonferroni correction for multiple testing: *ASF1A* and *IL7R* (Table 2, p<0.0022). These two genes showed a significant decrease in expression in samples from the long-lived families compared to controls, in concordance with the microarray results.

### Analysis of healthy offspring

We noticed that the effect sizes of the differential expressed genes in the comparison between offspring and controls were small. Since the offspring are composed of individuals who carry the longevity trait and those that do not, the detectable effect of the longevity trait is diluted in the comparison between offspring and controls. We hypothesize that the effect of the longevity trait will increase if we compare the same controls to a subfraction of offspring with the highest probability to age healthily and become long-lived. Therefore we selected the offspring with the most beneficial metabolic profile.

Offspring of long-lived parents exhibit as a group at least six beneficial serum characteristics including: low levels of glucose [28], [29], triglycerides [30], total cholesterol over HDL cholesterol ratio [31], [32], free triiodothyronine [33], and large low-density lipoprotein (LDL) particles [34], complemented by high adiponectin [35]. The beneficial metabolic profile is also reflected by the lower Framingham risk scores [36] which indicates a lower risk of cardiovascular disease over the course of 10 years.

We selected a subfraction of the offspring, consisting of the 5% beneficial tail of the distribution of each metabolic parameter, separately for men and women, resulting in 78 offspring which we consider the best proxy for the true long-lived cases. For these offspring *ASF1A* and *IL7R* gene expression was compared to that of 312 controls: this comparison resulted respectively in a 5.0% and 1.2% additional decrease of mean gene expression relative to the effect observed in the comparison between all offspring and controls (Table 3).

**Table 3:** Gene expression levels of *ASF1A* and *IL7R* in all as well as subfraction of offspring compared to controls.

			*ASF1A*		*IL7R*	
		n	Mean	p	Mean	p
Controls	All	312	1.85		6.18	
Offspring	All	332	1.65	**0.002** 2	5.98	**0.001** 2
	Subfraction^1^	78	1.57	**0.009** 3	5.91	**0.010** 3

Mean: relative expression in fold change to reference value.

1 : subfraction of offspring most probable to age healthily (for more details, see Materials and Methods),

2 : p value of comparison between controls and all offspring,

3 : p value of comparison between controls and subfraction of offspring (best 5% men and women per parameter taken together). Bold indicated p values are below the significant level of 0.05 after Bonferroni correction for multiple testing.

## Discussion

We have identified a transcriptional profile of 244 genes that represent a potential “longevity-signature”. In an extensive biological replication study of nonagenarians, middle-aged offspring and controls, we focused on a subset of 25 genes in RT-PCR experiments. An “aging-signature” was formed by the expression pattern of 21 genes. Two genes, *ASF1A* and *IL7R*, represented a “longevity-signature” since members of long-lived families expressed at middle-age a 1.14-fold and 1.12-fold lower level of these genes compared to controls respectively. The effect size of this association with longevity was stronger for offspring of nonagenarians with a beneficial metabolic profile characterized by 6 serum parameters and the Framingham Risk Score, which we considered to be the best proxy for the true long-lived case group. The decreased expression of these two genes is therefore likely to mark metabolic health and it might precede or even contribute to human longevity. ASF1A is a histone chaperone that is important in the remodeling of chromatin structure during replication, DNA repair, and cellular senescence [37]–[39]. Interestingly, histone acetylation is among the processes regulated by signaling through the IL7 receptor, which is required for development and maintenance of the immune system [40]. Our results may point at the importance of interactions between immune response, metabolic state, and epigenetic control for human aging and longevity.

The genome-wide expression analysis between nonagenarians and controls resulted in the identification of 2,953 probes associated with age. RT-qPCR replication experiments with a large sample size resulted in replication of 21 out of 25 genes identified by the microarray analysis as differentially expressed with age. Five of these genes (*LRRN3*, *ZNF331*, *ASF1A*, *MLLT3* and *SIDT1*) have previously been reported to associate with age in a microarray study of T cell mRNA [41], although this study had a relatively small number of samples (25 male subjects). The largest age-related effects observed in our study were for the *IFI27* and *LRRN3* genes. *IFI27* encodes interferon alpha-inducible protein 27 whose biological function is currently unknown. Leucine rich repeat neuronal 3 (*LRRN3*) is mainly involved in activation of MAPK activity and endocytosis [42] and, further supporting our findings, also showed the largest age-related decrease in expression in a large study on lymphocytes [16]. This “aging-signature” included decreased expression of several well-known genes involved in aging and lifespan, like *MYC*, *WRN* and *MTOR*. The MYC protein is a transcription factor that regulates transcription of specific target genes and overexpression of the *MYC* gene has been associated with a variety of cancers [43]–[45]. Defects in the *WRN* gene cause the Werner progeria syndrome, an autosomal recessive disorder characterized by premature aging and genetic variation in or near this gene have been associated with several age-related diseases and survival [46]–[49]. The *MTOR* gene encodes a serine/threonine kinase and its downregulation is associated with extended lifespan in model organisms [50]–[52] and elevated mTOR activity has been implicated in different forms of human cancer [reviewed in 53], [54]. We conclude from our data that, from all significantly differentially expressed genes at least 21 genes distinguish nonagenarians from middle-aged controls. In addition, our study is the first to include an extensive biological replication sample set validating these results.

The main cellular pathways that changed with age in the microarray dataset were ‘response to stimulus’ (including ‘immune response’ and ‘response to stress’), ‘signal transduction’, ‘gene expression’ and ‘protein binding’. These GO terms have previously been found to associate with aging, suggesting that these are systemic age-related processes. The only pathway that associated with longevity at middle-age in the offspring-control comparison was the ‘Rho protein signal transduction’ pathway, which is part of the GO term ‘signal transduction’. The Rho family of GTPases are small GTPases that regulate a wide variety of processes in the cell including growth, cytoskeletal organization, transcription and lipid metabolism [55], [56]. Rho signaling is regulated by the mTOR complex 2, a part of the mTOR pathway which is shown to influence lifespan and health. All associated pathways are general processes, indicating that regulation of the system seem to be an important process involved in aging and longevity.

Research into human familial longevity and healthy ageing is complex in the sense that there are no controls from the same birth cohort to compare to long-lived persons since such controls would have died twenty years ago. We investigated offspring as a proxy for the nonagenarian case group since these can be compared to controls from the same birth cohort. However, in the offspring the longevity phenotype will undoubtedly be diluted as compared to the nonagenarians, since only a part of the offspring will become long-lived and a part will age comparable to controls. The consequence of the dilution of the longevity cases is that effect sizes are underestimated. Indeed the effect size of the association with longevity increased when the healthiest offspring was compared to the controls. Future follow-up data on age of death will reveal which offspring carries the longevity phenotype.

In this study we investigated expression profiles in whole blood samples of participants because blood is an easily accessible tissue. This allows us to investigate the large sample sizes required to detect small effect sizes. An advantage of using blood compared to other tissues is that cell subsets can easily be measured and used to select samples or used as covariates in analyses whereas different cell subsets present in other tissues are difficult to quantify and can not be taken into account in any analyses. In our microarray study we selected samples from offspring and controls with similar cell counts. Furthermore, since aging affects the whole organism and since blood is in contact with all tissues, blood may reflect in part the physical health of the whole body. Disease state is mirrored by gene expression profiles in blood [summarized in 57], which at least partial overlap with expression profiles in other tissues [58]. Thus, although tissue-specific effects will undoubtedly be missed, investigating blood is valuable and practical for researching human aging.

In conclusion, we identified a transcriptional signature in whole blood consisting of 21 genes that repeatedly differentiated between nonagenarians and middle-aged controls. The expression level of two of these genes, *ASF1A* and *IL7R* marked familial longevity already in middle-age and the effect size was enhanced in the subset of longevity family members with a beneficial metabolic marker profile. Functional and longitudinal studies are necessary to establish which of these genes are true biomarkers for healthy ageing and which contribute causally to this trait. Our findings illustrate that gene expression changes occurring as a function of age may partly represent early detectable features of human longevity and healthy ageing.

## Materials and Methods

### Study population

The individuals investigated in this study are participants of the Leiden Longevity Study. The families participating in this study have at least two siblings with a minimum age for men of 89 years and for women of 91 years [59]. The offspring of these long-lived individuals, who have an increased potential to become long-lived individuals, were also included. In addition, the partners of the offspring were included as population controls of similar age and environmental exposures as the offspring, and as a young control group for the nonagenarian siblings. Blood samples were taken from all the participants. The Leiden Longevity Study was approved by the medical ethical committee of the Leiden University Medical Centre and all participants gave written informed consent.

### Sample collection and RNA preparation

One long-lived sibling, one of their offspring and the partner of the offspring were selected from 50 families for the current study (Table 1). These trios were randomly selected, but in such a way that age and gender were balanced between the groups and the age range for the offspring and partners was as large as possible. Additionally, individuals with outlying cell counts (beyond 3 SDs below or above the standard error of the mean) were excluded. From the 150 selected non-fasted individuals, peripheral blood was harvested using PAXgene™ tubes (Qiagen, Venlo, The Netherlands). The tubes were frozen and kept at −20°C for ∼3–5 years. After thawing at room temperature for at least 2 hours, total RNA was extracted from the approximately 2.5 ml of peripheral blood in each tube following the manufacturer's recommended protocol (PAXgene Blood RNA Kit Handbook, Qiagen, Venlo, The Netherlands). The quality and integrity of the total RNA was evaluated on the 2100 Bioanalyzer (Agilent Technologies, Amstelveen, The Netherlands) and the concentration was measured using a NanoDrop spectrophotometer (NanoDrop Technologies, Wilmington, DE, USA). Quality criteria included a 28S/18S ratio as measured by the Bioanalyzer of at least 1.2, and a total RNA yield of at least 3 µg.

### Oligonucleotide microarrays

The 150 samples that met the RNA quality criteria were hybridized onto 54k CodeLink Human Whole Genome Bioarrays (GE Healthcare, Bucks, UK, cat. No. 300026, currently of Applied Microarrays). cDNA synthesis, amplification, biotin labeling and hybridization onto the microarrays were performed according to the manufacturer's instructions using the Codelink iExpress reagent kit (cat. No. 67601000). The slides were scanned with a MicroArray Scanner G2505B scanner (Agilent Technologies, South Queensferry, UK) and the image was quantified with the CodeLink Expression software (version 4.2).

### Microarray data pre-processing

Raw intensities were background subtracted, set to 0.5 when results were negative and normalized using the Cyclic Loess method in the Codelink software package [60] of the Bioconductor R software [61] (http://www.bioconductor.org). After normalization, we used log_2_-transformed expression intensities for all subsequent analyses. Raw and normalized microarray data are stored in the GEO online database record GSE16717 in compliance to MIAME guidelines. A principal component analysis (PCA) was performed on all samples (GeneSpring software, Agilent Technologies, South Queensferry, UK) and hybridization date was identified as a confounding factor causing a deviation in the data, which was attributed to a scanner maintenance check during the measurements of the samples (data not shown). Therefore, all subsequent analyses were adjusted for hybridization date coded as the 14 days of hybridization as categorical variable, which was sufficient to adjust for this technically induced variation. Since samples were randomly hybridized, no confounding with group was present. None of the other tested parameters, like RNA quality, isolation date and time of blood draw appeared to be a significant confounder of the expression data.

### Probe annotation and filtering

The 54,243 probes on the CodeLink Bioarray were newly annotated to Entrez Gene ID's and GO identifiers in two steps. First, all probe sequences were mapped to Unigene and dbEst sequences with BLAT while allowing for, at most, one mismatching nucleotide. Subsequently, all Probe to Unigene annotations were transformed to probe to Entrez Gene ID and probe to GO ID annotations using Entrez Gene-to-Unigene and Entrez Gene-to-GO ID mappings available on the ftp server of NCBI. Probe-to-EST annotations were treated in a similar way, except that ESTs were first mapped to RefSeq Gene IDs by aligning ESTs to RefSeq exons using galaxy and the genomic alignments of ESTs and RefSeq genes from UCSC (hg18). All EST to RefSeq mappings with a sequence similarity >95% were maintained for further mappings of ESTs-to-Entrez Gene IDs using the Entrez Gene-to-RefSeq Gene ID mappings available at NCBI. All information used was downloaded in October 2008, using versions NCBI Build 36.1 or UCSC hg18.

Probes without a “Good” flag indicating that the mRNA is detected, as determined by the CodeLink Expression software, in at least 10% of the samples (7,034 probes) and/or probes without at least a known chromosome band location according to the new annotation (an additional 2,045) were excluded from the analysis, resulting in 45,164 remaining probes.

### Single gene analysis

All single gene analyses were performed using the Limma (Linear models for microarray data) package in R [62], [63]. To determine changes in expression levels of each probe with age, we used the following linear regression model:
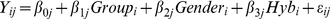
(1)where *Y_ij_* is the base 2 logarithm of the expression level of probe *j* in sample i, *Group_i_* is the group (0 for control or 1 for long-lived nonagenarian) of subject contributing sample *i*, *Gender_i_* corresponds to the gender of the *i*th sample (0 for male, or 1 for female), *Hyb_i_* is the categorical term of hybridization day on which the sample *i* was measured and ε*_ij_* represents an error term. The coefficients β_1*j*_ represents by how much gene expression increases between the groups, β_2*j*_ represents the change in expression for a female in comparison with a male sample, β_3*j*_ represents the change of expression across hybridization dates, and *β_0j_* represents the baseline regression level of the probe, for male control samples on the first hybridization date. Resulting p-values were adjusted for multiple testing using Benjamini and Hochberg's False Discovery Rate (FDR) method [64]. One-dimensional hierarchical clustering of probes was performed using GeneSpring (version GX 7.3.1) gene tree clustering, using Pearson correlation and average linkage.

To find longevity-related differences between offspring and controls, the following model was used:
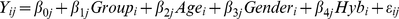
(2)where *Group_i_* corresponds to the offspring/control status of the *i*th sample (0 for control, 1 for offspring) and β_1*j*_ is the change of expression with group status. For each probe *j*, we determined the coefficient with respect to group status (β_1*j*_).

To test differences in biological aging rate in offspring and controls, we used the following model:
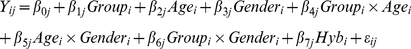
(3)where Group_i_*Age_i_ corresponds to the slope of expression with age of the *j*th probe set for offspring or controls, Age_i_*Gender_i_ corresponds to the slope of expression with age of the *j*th probe set for males or females, Group_i_*Gender_i_ indicates the interaction of group and gender of the *j*th probe set for the *i*th sample, β_4*j*_ represents the change of expression with age for offspring in comparison to control, β_5*j*_ represents the change of expression with age for a female in comparison with a male sample, β_6*j*_ represents the change of expression with gender between offspring and controls. The interaction between group and offspring in comparison to control (β_2*j*_ for the intercept, β_4*j*_ for the other groups) was determined and resulting p-values were corrected for multiple testing using the FDR method.

### Pathway analysis

The Globaltest methodology was designed to determine whether the common expression pattern of genes within a pre-defined set is significantly related to clinical outcome [21], [65], [66]. A generalized linear model is used to estimate a “Q-statistic” for each gene set, which describes the correlation between gene expression profiles, X, and clinical outcomes, Y. The Q-statistic for a gene set is the average of the Q-statistics for each gene in the set. The Globaltest method was used to perform pathway analysis on Model 1.

When performing pathway analysis on Model 3 we used GlobalAncova, which is a method similar to Globaltest suited for models including interaction terms [67], [68]. Resulting p-values from both methods were corrected for multiple testing using Holm's procedure for controlling the Family-Wise Error Rate (FWER) method [69].

### RT-qPCR

To confirm the accuracy of the measured expression profiles, we compared the expression level of 25 probes from the CodeLink Bioarrays with corresponding Taqman® assay (Applied Biosystems, Table 2). Samples included 18 nonagenarians, 16 offspring and 21 controls that have been measured on the microarray and additional novel replication samples of randomly chosen 61 nonagenarians, 316 offspring and 291 controls. Reverse transcription was performed by using total RNA from blood of in total 723 samples, excluding individuals with outlying cell counts (outside 3SD of the mean), which passed QC and processed with the First Strand cDNA Synthesis Kit according to the manufacturer's protocol (Roche Applied Science). cDNA was amplified using the DNA Engine Tetrad® 2 Peltier Thermal Cycler (Bio-Rad). qPCR was then performed with the Taqman® method using the Biomark™ 48.48 and 96.96 Dynamic Arrays (Fluidigm). Relative gene expressions of the BioMark™ Array data were calculated by using the 2^−ΔΔCt^ method, in which Ct indicates cycle threshold, the fractional cycle number where the fluorescent signal reaches detection threshold [70]. YKT6 was used as internal control and commercially available human total reference RNA (Clontech Laboratories, Mountain View, CA, USA) as reference RNA. Differences in expression level between long-lived siblings, their offspring and the partners of their offspring were assessed using linear regression. In these analyses, expression level was the dependent variable and the two groups of individuals (either nonagenarians vs. controls or offspring vs. controls) were included in the model as a categorical variable together with age (in offspring vs. controls only) and gender and their interaction as covariates. To take into account dependencies within sibships, robust standard errors were used, i.e. the variance was computed from the between family variation. P-values were also based on these robust standard errors. Analyses were performed using the software package STATA/SE 11.0 (DPC Software, StataCorp 2009).

### Analysis of healthy offspring

To further investigate the candidate genes, their expression level was again tested for association with longevity, but only including the offspring with most beneficial profile of seven published longevity markers: a low level of non-fasted serum glucose (mmol/L) [71], [72], triglycerides (mmol/L) [73] and free triiodothyronine levels (pmol/L) [74], a small ratio of total cholesterol (mmol/L) over HDL cholesterol (mmol/L) [75], [76], small low-density lipoprotein (LDL) particle sizes (nm) [77], a high level of adiponectin (mg/L) [26], and a low Framingham risk score (FRS) which is based on the factors age, sex, total cholesterol level, HDL cholesterol level, systolic blood pressure (mm/Hg), and whether the person smokes [78]. The FRS is a well known test reflecting the risk of cardiovascular disease over the course of 10 years.

All serum measurements were performed with fully automated equipment. For glucose, triglycerides, total cholesterol, HDL-cholesterol, adiponectin and free triiodothyronine, the Hitachi Modular or the Cobas Integra 800, both from Roche, Almere, the Netherlands were applied. CVs of these measurements were all below 5%. Lipoprotein particle sizes have been analyzed in 165 families from the Leiden Longevity Study using a 400-MHz proton NMR analyzer at LipoScience.

To select that subfraction of offspring with the highest probability to age healthily and become long-lived because of their metabolic risk profile in middle-age, we identified the subjects, separately for men and women, within the lower 5% tail of the distribution for glucose, triglycerides, free triiodothyronine, ratio of total cholesterol over HDL cholesterol and the FRS. Additionally we identified for LDL particle size and adiponectin those subjects, separately for men and women, within the upper 5% tail of the distribution. This resulted in a total of 78 offspring out of 332 from which gene expression levels of *ASF1A* and *IL7R* was compared to the levels in 312 controls using linear regression. In this analysis, expression level was the dependent variable and the two groups of individuals (offspring vs. controls) were included in the model as a categorical variable together with age and gender and their interaction as covariates. To take into account dependencies within sibships, robust standard errors were used, i.e. the variance was computed from the between family variation. P-values were also based on these robust standard errors. Analyses were performed using the software package STATA/SE 11.0 (DPC Software, StataCorp 2009).

## Supporting Information

Figure S1 **Distribution of expression intensities of 2,953 age-related probes.** The x-axis indicates average log2 expression intensities of all samples; the y-axis indicates the log2 fold change between nonagenarians and controls. Black circles represent all 45,164 probes; red filled dots represent the 2,953 age-related probes with a FDR≤0.05.
(TIF)

Table S1 **Significant gene expression changes with chronological age and/or familial longevity.** A total of 2,953 probes whose expression level changed significantly between controls and long-lived nonagenarians (regression modeling with FDR multiple testing correction) are shown. The table also indicates the chromosomal location of the probe. “FC” indicates the fold change between groups, a FC above 1 indicates increased expression in nonagenarians compared to controls, and a FC below 1 indicates decreased expression in nonagenarians compared to controls. MULTIPLE indicates a probe annotated to more than three chromosomal locations.
(XLS)

Table S2 **GO terms found significantly differentially expressed between nonagenarians and controls.** Globaltest pathway analysis resulted in 109 Gene Ontology terms of which the expression of the involved probes differed between long-lived nonagenarians and controls (FWER≤0.05). Gene Ontology categories include biological process (BP), molecular function (MF) and cellular component (CC).
(XLS)

Table S3 **List of probes of which the gene expression changes with age significantly differed between offspring and controls.** A total of 360 probes whose expression level changed significantly with age between offspring and controls (regression modeling with FDR multiple testing correction) are shown. “FC” indicates the fold change between offspring and controls per 10 years; a FC above 1 indicates a larger slope of expression as a function of age in offspring, a FC below 1 indicates a larger slope of expression as a function of age in the controls. MULTIPLE indicates a probe annotated to more than 3 chromosomal locations.
(XLS)

Table S4 **RT-qPCR results of replication samples only.** RT-qPCR results of replication samples only are shown. “FC” indicates the fold change between groups; a FC above 1 indicates an increase in expression and a FC below 1 indicates a decrease in expression compared to the controls. “p” indicates the unadjusted p values. Bold indicate p values are below the significant level of 0.05 after Bonferroni correction for multiple testing.
(DOC)
