# Correction: Transcriptional Profiling of Human Familial Longevity Indicates a Role for *ASF1A* and *IL7R*

**DOI:** 10.1371/annotation/d4d3b1fb-35e0-4c4d-89bc-e6a839504e54

**Published:** 2012-05-10

**Authors:** Willemijn M. Passtoors, Judith M. Boer, Jelle J. Goeman, Erik B. van den Akker, Joris Deelen, Bas J. Zwaan, Ann Scarborough, Ruud van der Breggen, Rolf H. A. M. Vossen, Jeanine J. Houwing-Duistermaat, Gert Jan B. van Ommen, Rudi G. J. Westendorp, Diana van Heemst, Anton J. M. de Craen, Andrew J. White, David A. Gunn, Marian Beekman, P. Eline Slagboom

A funding source was omitted from the published funding statement. The authors would like to add: The research leading to these results has received funding from the European Union's Seventh Framework Programme (FP7/2007-2011) under grant agreement n° 259679 (IDEAL). 

