# Supplementary material for: Transcriptional Profiling of Human Familial Longevity Indicates a Role for ASF1A and IL7R
Source: PLoS One. 2012 Jan 11;7(1):e27759. doi: 10.1371/journal.pone.0027759 (PMC3256132; doi:10.1371/journal.pone.0027759)
Supplement: Table S4 — RT-qPCR results of replication samples only. RT-qPCR results of replication samples only are shown. “FC” indicates the fold change between groups; a FC above 1 indicates an increase in expression and a FC below 1 indicates a decrease in expression compared to the controls. “p” indicates the unadjusted p values. Bold indicate p values are below the significant level of 0.05 after Bonferroni correction for multiple testing. (DOC) [file pone.0027759.s005.doc]

|  | |  | Nonagenarians vs controls | | Offspring vs controls | |
| --- | --- | --- | --- | --- | --- | --- |
|  | | N = | 58 | 281 | 305 | 281 |
| Gene name | | Assay | FC | p | FC | p |
|  | *Top genes* |  | | | | |
| IFI27 | | Hs01086373_g1 | 1.47 | **7.3*10^-4^** | NA | NA |
| LRRN3 | | Hs00539582_s1 | 0.57 | **<10^-6^** | NA | NA |
| ZNF331 | | Hs00218578_m1 | 0.93 | **0.002** | 0.99 | 0.339 |
|  | *Cell aging associated genes* | | | | | |
| ADAMTS5 | | Hs00199841_m1 | 1.01 | 0.037 | 1.00 | 0.919 |
| ASF1A | | Hs01011627_m1 | 0.85 | **3.7*10^-5^** | 0.88 | 0.004 |
| CCR6 | | Hs01890706_s1 | 0.68 | **<10^-6^** | 1.06 | 0.273 |
| CD248 | | Hs00535586_s1 | >10 | 0.321 | 1.02 | 0.874 |
| CDK6 | | Hs00608037_m1 | 0.95 | 0.228 | 0.94 | 0.010 |
| ENO2 | | Hs00157360_m1 | 0.99 | **7.0*10^-6^** | 0.99 | 0.064 |
| FLT3LG | | Hs00181740_m1 | 0.89 | 0.159 | 0.53 | 0.094 |
| HK3 | | Hs01092843_g1 | 1.14 | **8.0*10^-6^** | 0.99 | 0.774 |
| IL7R | | Hs00902338_g1 | 0.76 | **<10^-6^** | 0.89 | **0.002** |
| LEF1 | | AI6Q1P7 | 0.61 | **<10^-6^** | 0.96 | 0.414 |
| MLLT3 | | Hs00971090_m1 | 0.80 | **<10^-6^** | 0.95 | 0.085 |
| MTOR (FRAP1) | | Hs00234508_m1 | 0.97 | **2.0*10^-4^** | 0.99 | 0.314 |
| MYC | | Hs00153408_m1 | 0.78 | **2.0*10^-6^** | 0.97 | 0.338 |
| NOLC1 | | Hs01102319_g1 | 0.87 | **1.2*10^-5^** | 0.95 | 0.092 |
| NR3C2 | | Hs00230906_m1 | 0.77 | **<10^-6^** | 0.95 | 0.075 |
| RUVBL2 | | AI7ZZWF | 0.81 | 0.006 | 1.07 | 0.461 |
| SIDT1 | | Hs00214475_m1 | 0.73 | **5.5*10^-4^** | 0.88 | 0.167 |
| SMAD3 | | Hs00706299_s1 | 0.87 | **<10^-6^** | 0.99 | 0.862 |
| SMYD5 | | Hs00300181_m1 | 0.93 | **2.7*10^-5^** | 0.98 | 0.189 |
| TCF12 | | Hs00918972_m1 | 0.90 | **1.1*10^-5^** | 0.96 | 0.085 |
| TCF4 | | Hs00972428_g1 | 0.89 | **0.001** | 0.99 | 0.849 |
| WRN | | Hs02561119_s1 | 0.77 | **<10^-6^** | 1.02 | 0.770 |
